# Enhanced Sympathetic Arousal in Response to fMRI Scanning Correlates with Task Induced Activations and Deactivations

**DOI:** 10.1371/journal.pone.0072576

**Published:** 2013-08-15

**Authors:** Markus Muehlhan, Ulrike Lueken, Jens Siegert, Hans-Ulrich Wittchen, Michael N. Smolka, Clemens Kirschbaum

**Affiliations:** 1 Institute of Clinical Psychology and Psychotherapy, Department of Psychology, Technische Universität Dresden, Dresden, Germany; 2 Chair of Biopsychology, Department of Psychology, Technische Universität Dresden, Dresden, Germany; 3 Section of Systems Neuroscience, Department of Psychiatry and Psychotherapy, Technische Universität Dresden, Dresden Germany; 4 Neuroimaging Center, Department of Psychology, Technische Universität Dresden, Dresden, Germany; National Research & Technology Council, Argentina

## Abstract

It has been repeatedly shown that functional magnetic resonance imaging (fMRI) triggers distress and neuroendocrine response systems. Prior studies have revealed that sympathetic arousal increases, particularly at the beginning of the examination. Against this background it appears likely that those stress reactions during the scanning procedure may influence task performance and neural correlates. However, the question how sympathetic arousal elicited by the scanning procedure itself may act as a potential confounder of fMRI data remains unresolved today. Thirty-seven scanner naive healthy subjects performed a simple cued target detection task. Levels of salivary alpha amylase (sAA), as a biomarker for sympathetic activity, were assessed in samples obtained at several time points during the lab visit. SAA increased two times, immediately prior to scanning and at the end of the scanning procedure. Neural activation related to motor preparation and timing as well as task performance was positively correlated with the first increase. Furthermore, the first sAA increase was associated with task induced deactivation (TID) in frontal and parietal regions. However, these effects were restricted to the first part of the experiment. Consequently, this bias of scanner related sympathetic activation should be considered in future fMRI investigations. It is of particular importance for pharmacological investigations studying adrenergic agents and the comparison of groups with different stress vulnerabilities like patients and controls or adolescents and adults.

## Introduction

It has been repeatedly shown that magnetic resonance imaging (MRI) examinations can trigger stress responses [1]–[9]. In 1988 Brennan and colleagues were the first to report that routine care patients undergoing MRI investigations, showed ‘sympathetic symptoms of adrenergic discharge’ prior to the scanning procedure [3]. However, a direct measurement of sympathetic changes throughout the session was conducted over a decade later, possibly because MR compatible record systems were not available until then. In 2010 Chapman and colleagues reported that heart rate and anxiety ratings showed the highest values at the beginning of a spectroscopic MR scanning procedure, steadily decreased over time and peaked again at the end of the session [5]. Changes in sympathetic activity however, have been frequently shown to interact with performance and neural correlates of cognitive and emotional processes [10] like motor inhibition [11], executive functions [12], processing of concealed information [13], selective attention [14], [15] or threat perception [16], [17]. Moreover, regions of the default mode network, that are regularly deactivated during task performance [18] have also been shown to be affected by sympathetic changes. Recent evidence suggests that increased sympathetic activity during painful stimulation correlates with the task induced deactivation (TID) [19], [20]. Against this background it is plausible that sympathetic changes evoked by the scanning session itself can act as potential confounders in fMRI experiments. Consequently, several study designs appear to be particular vulnerable to a bias caused by changes in sympathetic activation levels. A major problem of fMRI experiments is the observed habituation to the scanner environment over time [2], [9], which may result in intraindividual fluctuations of sympathetic activation throughout the scanning session [21], [22]. Higher sympathetic reactions at the beginning of an fMRI experiment could thus account for intersession fluctuations that decrease the reliability of neural and behavioural data obtained by a broad range of frequently used fMRI paradigms [23]–[25]. In a prior study conducted in our lab [1] we were able to show that salivary alpha amylase (sAA) secretion, a valid indicator for sympathetic activation [26], changed significantly during an fMRI session. Comparable to the results reported by Chapman et al. [5] we found the highest peak at the beginning of the experiment and a second at the end of the examination. Preliminary results revealed a positive correlation between sAA levels and thalamic activity. In the present study we reanalysed the data in view of the variability of neural and behavioural data in a larger sample. A task was performed twice during fMRI scanning to test for intersession differences. The task required specific motor preparation to different expected or unexpected time points. This paradigm was associated with an activation of motor areas in anticipation of the upcoming target [27]. As started above, prior studies have revealed that sympathetic arousal correlates with task induced activations and deactivations. Based on these findings we hypothesized an association between changes of sAA secretion and task induced activation and task induced deactivation (TID). Furthermore, we predicted a stronger effect during the first part of the experiment, were the most pronounced changes in sAA were observed [1], compared to the second part.

## Methods

### Subjects and Procedure

Forty-seven, scanner-naive, right handed students from the Technische Universität Dresden were recruited using flyers and public announcements. All subjects had normal or corrected to normal vision. Exclusion criteria included smoking, oral contraceptives, body mass index below 18 or above 26, prior (MRI, PET or CT) scanning experience, any history of acute or chronic medical disease or failure to meet MRI compatibility. Subjects received course credit or €10 for participation. All subjects gave their written informed consent. The study was conducted in accordance with the Declaration of Helsinki and approved by the Ethics Committee of the Technische Universität Dresden. Subjects arrived between 11:00 am and 5:00 pm at the Neuroimaging Center and were asked to come well rested to testing and were also requested not to eat or drink one hour before the experiment. To ensure that all participants had adequate levels of blood glucose they received 2 dl grape juice immediately after the first saliva sample. Six subjects were excluded from further analysis due to response to catch-trials, see below, (n = 2) and incomplete biochemical data (n = 4). Four subjects were additionally excluded from the fMRI analysis due to excessive movement parameters > 3 mm resulting in a total fMRI dataset of n = 37 (male: n = 31; mean age  = 24.06 (± 2.39); female: n = 6; mean age  =  23.17 (± 3.19)).

After entering the lab, subjects were informed about the study protocol and MRI procedure. Then subjects performed a training session of the task for 20 min to control for further learning effects during the fMRI examination. Thereafter subjects were brought into the examination room and prepared for scanning. MRI data were acquired in the following order: functional run 1 (15 min); structural scan (10 min); functional run 2 (15 min). Both functional runs were identical. Saliva samples were obtained before and after the training runs, after the MRI preparation (immediately before entering the scanner) after run 1 and before and after run 2. MRI staff was held constant for all subjects. For a detailed description of the study protocol please see Muehlhan et al., [1].

### Task

A temporal cued target detection task similar to that used by Coull, et al. (2000) [27] was applied. The task required to direct attentional resources to a particular time point. Baseline stimuli (BL) were two concentric circles with a small centred fixation cross presented foveally on a black background (see figure.? 1). Each trial started with a brightening (100 ms) of one of the circles indicating either a 600 ms (inner circle) or a 1400 ms (outer circle) cue-target interval (CTI) followed by the appearance (100 ms) of a large cross (target) superimposed over the BL. Participants were required to indicate detecting the target by pressing a button with the right index finger as fast as possible. A total of 400 trials per run were presented in a pseudo-randomized order, 235 valid trials, 55 invalid trials, 10 catch trials and 100 null events as a respective baseline [28]. The randomized presentation of different trials and null-events leads to variable stimulus onset asynchronies preventing stimulus-response predictability. During valid trials participants were required to orient attention to a particular time point 600 ms (short CTI) or 1400 ms (long CTI) after the presentation of the cue stimulus. During the invalid trials the CTI was reversed. This required a re-orientation of attention to the unexpected appearance of the target. During catch-trials, only the cue but not the target was shown. Subjects who reacted on the catch-trials more than three times were excluded from the fMRI analysis. Each trial lasted 2000 ms.. Every tenth trial the paradigm was synchronized with the fMRI-TTL pulse. The paradigm was programmed using Presentation® 11.3 (Neurobehavioral Systems, Inc., CA). Stimuli were presented on video goggles (VisuaStim Digital, Northridge, California). Behavioural responses were acquired using a MRI compatible response Box (LUMItouch™, Photon Control Inc. Burnaby, BC, Canada).

**Figure 1:**
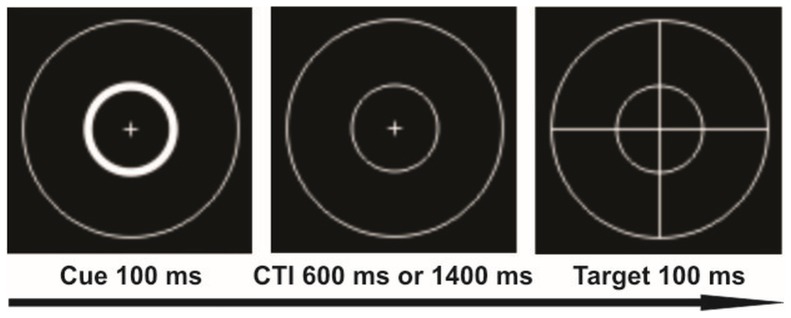
Schematic view of the target detection task. The example illustrates the brightening of the inner circle (cue) indicating a short cue target interval (CTI). After the cue stimulus a CTI of either 600 ms (expected) or 1400 ms (unexpected) followed, than the target appeared (large cross). Long CTIs’ were cued by brightening of the outer circle.

### Saliva sampling and analysis

Salivettes® ‘code blue’ (Sarstedt, Nümbrecht, Germany) with synthetic swabs were used for saliva sampling. Subjects were instructed to gently chew until the swabs were soaked with saliva. During scanning session, subjects lied on the MRI table and were put back in the home position. Swabs were handed to the subject by an assistant to avoid any movement. Samples were stored at –20°C until assayed in the biochemical laboratory. The measurement of sAA activity in the saliva samples was performed as previously described by Muehlhan et al. [1].

### MRI Data Acquisition

MRI images were acquired using a 3-Tesla Trio-Tim MRI whole-body scanner (Siemens, Erlangen, Germany). A standard 12 channel head coil and standard headphones were applied. In each functional run, 411 volumes of 38 axial slices with voxel size of 3×3×3 mm (1 mm gap) were acquired sequentially. Each slice had a matrix size of 64×64 voxels resulting in a field of view of 192 mm. Functional measurements were obtained using a T2* weighted gradient echo planar images (EPI) sequence (repetition time (TR) 2200 ms, echo time (TE) 25 ms, flip angle α = 80°). Structural images were obtained by using a Magnetization Prepared Rapid Gradient Echo Imaging (MPRAGE) sequence (TR 1900 ms, TE 2.26 ms, flip angle α = 9°).

### Data analysis

#### Physiological data

A one way ANOVA was performed to calculate changes in sAA over the six sampling points. Subsequent pairwise comparisons were used to identify significant changes between the sampling points. Greenhouse-Geisser adjustments were applied where appropriate. For all significant changes we calculated the percentage sAA change to account for individual variability in initial values. In detail, we calculated the percentage sAA change immediately prior to scanning ((SP3-SP2)/SP2)*100, the percentage sAA change before ((SP5-SP4)/SP4)*100 and during run 2 ((SP6-SP5)/SP5)*100. Since prior results revealed that some but not all subjects showed a sympathetic response in response to the scanner setting [1] we calculated the number subjects who showed a sAA increase, and the number of those who showed a sloping sAA secretion.

#### Behavioural data

Mean reaction times (RT) were calculated for every subject. RTs shorter than 50 ms and longer than 600 ms were counted as errors and were not considered in further analysis [27]. A one way ANOVA was used to calculate RT differences between the training session and the two task executions during the fMRI session. Moreover, Pearson correlations were performed to calculate the associations between RT means and percentage change of sAA. Because two subjects performed poorly (below 85% correct), compared to the high grade of accuracy from the entire group, associations between the percentages of correct responses and percentage change of sAA were calculated by using non-parametric Spearman correlations.

#### fMRI data

FMRI data were analysed using SPM8 (Welcome Trust Centre for Neuroimaging, UCL, London, UK). Prior to pre-processing, the first four scans were excluded from analysis to avoid T1 equilibration effects. The remaining functional scans of both runs were spatially realigned and unwarped to correct for interscan movement. Acquisition times were corrected by setting the reference to slice 16 (middle slice). Structural images were registered to the functional scans and all volumes were normalized to the MNI (Montreal Neurological Institute, Quebec, Canada) reference brain by applying a unified tissue segmentation and normalization algorithm. A smoothing kernel of 8 mm full-with half-maximum was used to accommodate interindividual anatomical variability. At the first level regressors were built for the effects of interest (valid and invalid trails) and the effects of no interest (catch trials) for run 1 and run 2 respectively. All regressors were modelled as single events and time locked to the onset of the cue. A 128 sec. high-pass filter was used to remove non-physiological slow signal shifts. The general linear model was used to calculate regression coefficients (beta values) for the regressors and each voxel. For the fixed effects a full factorial design was used to calculate the task induced activation and deactivation (run 1 and run 2). In the next step one sample t-tests for run 1 and run 2 were calculated for the main effect of the task and the percentage sAA change was integrated as a covariate. To identify brain regions that were influenced by sympathetic changes during the task, conjunction-null analyses [29] were used. The conjunction of task induced activation and percentage sympathetic changes, as well as the conjunction of the task induced deactivation (TID) and percentage sympathetic changes were calculated for both runs respectively. The statistical threshold was set to p<0.005 with a minimum cluster size of k = 30 voxels which has been discussed as a desirable threshold for imaging parameters [30]. The conjunction analyses revealed five separate cluster for the task induced activation and four separate clusters for TID. The resulting clusters were used to build binary masks. The mean beta values from each of these masked regions were extracted by using rfx_plot [31]. Finally, subsequent correlational analyses and scatter plots were included for illustrative purposes. Correlations between the extracted mean beta values and relative sAA increase as well as correlations between the mean beta values and behavioural data (RTs and correct responses) were performed using the statistical software package SPSS 19. Associations between RTs and relative sAA increase or beta values were calculated using Pearson correlations. Non-parametric Spearman correlations were used for the analyses of association between correct responses and relative sAA increase or beta values.

## Results

### Salivary alpha amylase

ANOVA results indicated significant changes of sAA during the six sampling points [F (3, 105)  =  3.902 p = 0.012, η = 0.098]. Subsequent pairwise comparisons revealed no significant sAA difference before and after the training session outside the scanner (SP1, SP2: p = 0.383) indicating no sympathetic activation that could be attributed to the task performance itself. Immediately prior to scanning, however, sAA rose significantly (SP2 < SP3; p = 0.010), up to its peak: SP3 > SP2; SP5; SP6; p<0.05 and remained at high levels during run 1: SP3, SP4: 0.286. Then a significant decrease was observed while the structural images were acquired (SP4 > SP5: p = 0.002) followed by a second increase at the end of the examination (SP5 < SP6; p = 0.009) (see figure? 2). We further calculated the number of subjects who showed an sAA increase prior to the first and during the second run. Immediately prior to scanning n = 24 subjects (64.9%) showed an increase of sAA secretion and for n = 13 (35.1%) a decrease was observed. At the end of the scanning session n = 27 subjects (72.9%) showed an increase of sAA secretion and n = 10 (27.1%) a decrease.

**Figure 2:**
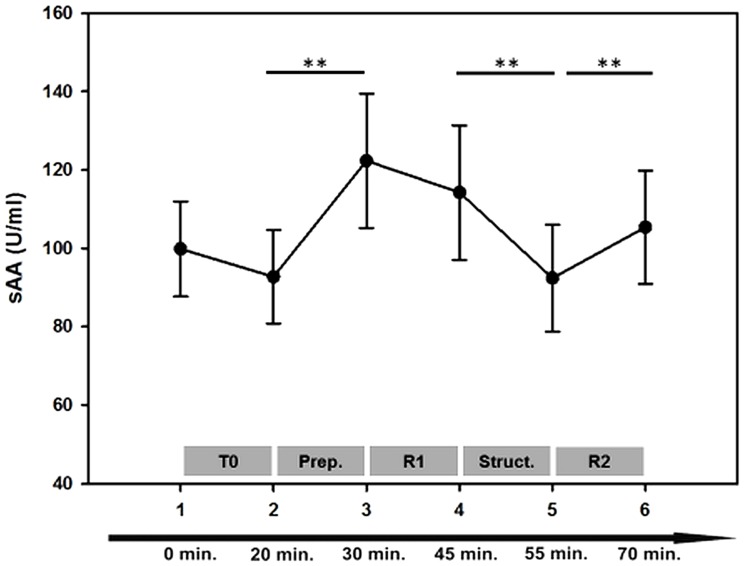
Salivary alpha-amylase (sAA) profile over the six sampling points. Error bars indicate SEM. T0  =  training phase; Prep.  =  MRI preparation (subjects were placed on the MRI table, get goggles, headphones, headcoil, ect.); R1  =  Run 1; Struct.  =  high resolution structural scan; R2  =  Run 2. **p<0.01.

### Behavioural data

#### Reaction times

RTs changed significantly between the training session, run 1 and run 2 [*F* (2, 66)  =  3.694, *p* = 0.030, η = 0.101]. Subsequent pairwise comparisons yielded significant differences between the training session and the both runs (run1 and run2) p<0.05. RTs of run 1 and run 2, however, did not differ significantly p = 0.660. (see table? 1) The percentage sAA increase during the preparation phase was negatively correlated with the mean RTs during run 1 r = –0.346; p = 0.034 (see figure? 3a). During run 2 neither the percentage sAA decrease during the structural scan nor the percentage sAA increase during run 2 showed significant associations with the RTs.

**Figure 3:**
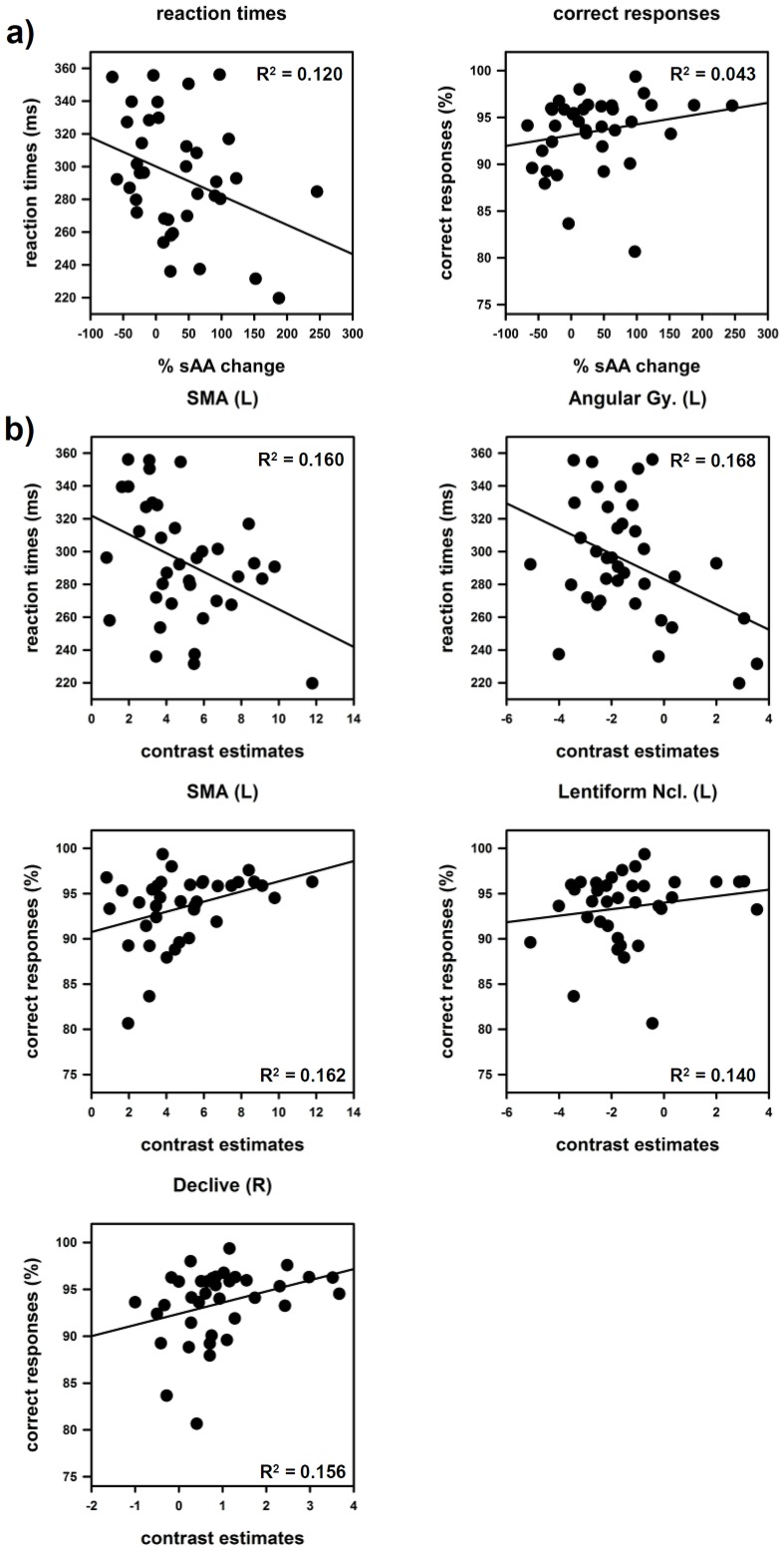
Scatter plots and coefficients of determination R^2^ of salivary alpha-amylase (sAA), brain activation/deactivation and behavioral data. a) Associations between reaction times (RT)/correct responses during run 1 and percentage sAA increase immediately prior to scanning. b) Associations between reaction times (RT)/correct responses and extracted mean beta values. Task induced activation: supplementary motor area (SMA), lentiform nucleus and declive. Task induced deactivation: angular gyrus.

**Table 1:** Mean reaction times and accuracy data.

	RT (SD)	% correct (SD)
Training	306.21 (35.41)	94.74 (3.19)
Run 1	293.87 (36.19)	93.50 (3.92)
Run 2	290.16 (39.28)	92.21 (5.71)

#### Accuracy

Subjects performed the task with a high grade of accuracy. The analyses of accuracy rates revealed significant changes between the training session and the two runs [*F* (2, 64)  = 7.404, *p*<0.001, η = 0.188]. Subsequent pairwise comparisons showed that subjects performed the task with the highest accuracy during the training phase. After entering the scanner, accuracy rates decreased significantly T0 > R1, R2, p  =  but did not differ between run 1 and run 2 (see table? 1) The correlation analyses revealed significant positive correlations r = .329; p = 0.046 between the percentage sAA increase prior to scanning and the accuracy rates during run 1 (see figure? 3a) No significant correlations could be found during run 2.

### fMRI data

#### Task induced activation and deactivation

As can be seen in figure? 4, the task activated a large network of several cortical and subcortical regions that peaked over the following structures: areas associated with motor preparation and motor performance: the left supplementary motor area (SMA), the left precentral gyrus, the left putamen and a cluster in the right cerebellar tonsil; areas related to motor inhibition: inferior frontal gyrus (IFG) and regions that are involved in visual processing: left and right middle occipital gyri (MOG) and attention: the left and superior parietal lobules (SPL), and the left thalamus. The analysis of the TID yielded regions centered over the following structures: right cuneus, left angular gyrus, right precentral gyrus, right and left middle temporal gyri (MTG), right and left Insulae, right and left lingual gyri, left inferior frontal gyrus (IFG), medial frontal gyrus (MFG) and superior frontal gyrus (SFG) and the left precuneus. Peak voxel, sub regions and cluster sizes are shown in table? 2.

**Figure 4:**
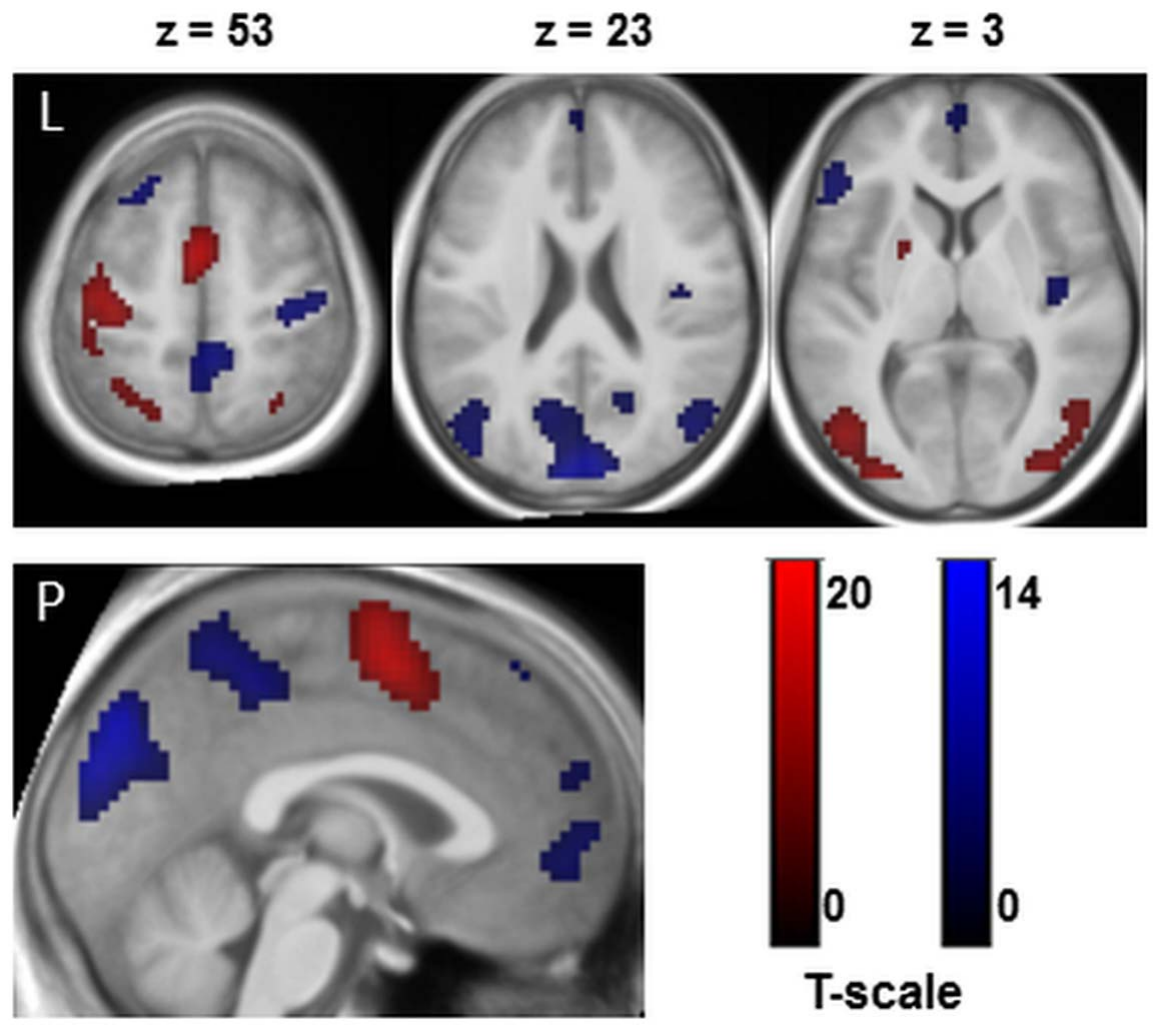
Task induced activation (red) and deactivation (blue). Clusters are presented on three axial slices and a medial slice of an anatomical spatially normalized mean image of all subjects. The statistical threshold was set to T≥10 for activation and T≥4 for task induced deactivation with a minimum cluster size of k = 10.

**Table 2:** Peak voxel and sub regions from the task induced activation and deactivation.

L/R	Brain Region	T value	cluster size k	coordinates		
				x	y	z
*Task induced activation*						
L	SMA	18.19	295	–6	2	58
L	Mid. cingulate	10.31		–9	8	37
L	Precentral Gy.	17.57	223	–39	–19	52
L	Postcentral Gy.	14.89		–57	–22	46
L	Precentral Gy.	12.32		–39	–4	52
R	MOG	17.51	648	33	–91	–2
R	Declive	13.83		36	–55	–23
R	Declive	13.66		30	–61	–20
L	MOG	16.94	611	–30	–91	–5
L	MOG	14.84		–42	–70	1
L	MOG	14.19		–30	–88	13
L	SPL	13.65	66	–27	–49	49
L	IPL	10.87		–35	–40	40
R	Tonsil	11.93	33	21	–61	–47
L	Lentiform Ncl.	11.51	24	–24	5	1
L	Thalamus	11.46	14	–12	–19	7
L	IFG	11.06	16	–60	5	28
L	Precentral Gy.	10.24		–54	2	34
R	SPL	11.04	10	33	–55	52
*Task induced deactivation (TID)*						
R	Cuneus	12.42	754	9	–88	28
L	Cuneus	10.04		–3	–88	19
L	Cuneus	8.80		–6	–79	31
L	Angular Gy.	11.58	227	–45	–79	31
R	Precentr. Gy.	9.78	673	45	–22	61
R	Precentr. Gy.	7.41		36	–19	46
R	Postcentr. Gy	7.04		30	–34	70
R	MTG	9.08	133	48	–78	28
R	Insula	7.90	184	42	–16	16
R	Insula	6.45		39	–13	1
L	IFG	6.97	98	–51	32	4
L	Lingual Gy.	6.76	44	–12	–70	–5
R	Lingual Gy.	6.70	33	12	–73	–2
L	IFG	6.26	59	–30	32	–17
L	MFG	5.94		–27	29	52
L	MFG	5.80		–27	35	45
L	SFG	5.40		–18	29	58
L	MTG	5.77	17	–63	–49	–8
L	Insula	5.31	11	–39	–19	1
L	Insula	5.22	45	–27	14	–20
L	MFG	5.17	63	–9	32	–11
R	ACC	5.15		6	26	–11
L	Lingual Gy.	5.07	69	–15	–46	–11
L	Parahippocampal	4.84		–24	–43	–8
L	Fusiform Gy.	4.68		–21	–34	–17
L	MFG	5.00	11	–6	56	–5
L	Precuneus	4.75	15	–9	–49	61

Peakvoxel based on a voxelwise T≥10 for activation and T≥4 for TID with a minimum cluster size of k = 10. SMA: supplementary motor area; MOG: middle occipital gyrus, MTG: middle temporal gyrus, MFG: medial frontal gyrus, IPL: inferior parietal lobule, IFG: inferior frontal gyrus, SFG: superior frontal gyrus, SPL: superior parietal lobule.

#### Conjunction of sympathetic arousal and the task induced activation

The conjunction analysis revealed five regions that were activated by the task in run 1 and that were additionally associated with the percentage sAA increase immediately prior to scanning (see figure? 5). All regions were parts of the motor system: left SMA: r = .535, p = 0.00032; left precentral gyrus: r = .542, p = 0.00026, left lentiform nucleus: r = .575, p = 0.00010, right lentiform nucleus: r = .541, p = 0.00027 and right declive: r = .578, p = 0.00009. Other regions activated by the task, such as middle occipital lobes or superior parietal lobules were not associated with sAA changes. Please see table? 3 for details about sub regions and cluster sizes. Note that all observed effects were restricted to the first run. During the second run no significant effects between sympathetic changes and task related activity could be found.

**Figure 5:**
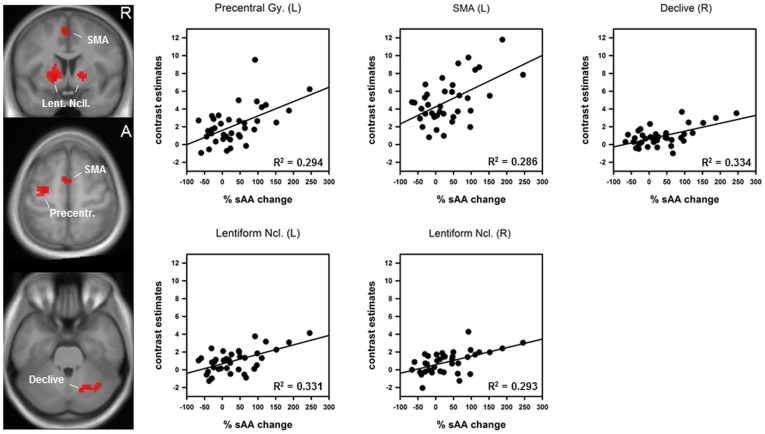
Conjunction of the main effect of the task in run 1 and percentage sAA increase immediately prior to scanning. *Left:* coronal and axial slices of an anatomical spatially normalized mean image of all subjects. Colour blobs indicate regions that are activated by the task and influenced by percentage salivary alpha-amylase (sAA) increase. *Right:* Scatter plots and coefficient of determination R^2^. SMA: supplementary motor area; Lent. Ncll.: lentiform nuclei.

**Table 3:** Peak voxel and sub regions from the conjunction analysis of task induced activation during run 1 and relative sAA increase immediately prior to scanning.

L/R	Brain Region	T value	cluster size k	coordinates			r =	p =
				x	y	z		
L	Lentiform nucleus	4.14	188	–15	5	–2	.575	.00010
L/R	Thalamus	3.52		0	–10	4		
L	SMA	3.63	47	–3	2	55	.535	.00032
R	Lentiform nucleus	3.59	40	15	8	–2	.541	.00027
L	Precentral Gy.	3.53	64	–27	–10	58	.542	.00026
L	Precentral Gy.	3.41		–33	–16	67		
R	Declive	3.33	55	24	–64	–29	.578	.00009
R	Culmen	3.19		36	–61	–32		
R	Declive	3.07		18	–70	–26		

Peakvoxel from the conjunction analysis are based on a voxelwise p<0.005, uncorrected and a minimum cluster size of k = 30. SMA: supplementary motor area.

#### Conjunction of sympathetic arousal and TID

The conjunction analyses yielded four regions that showed a deactivation during run 1 and that were additionally associated with the percentage sAA increase immediately prior to scanning (see figure? 6). The higher the sAA increase the lower the deactivation in the following regions: left angular gyrus comprising parts of the precuneus: r = .558, p = 0.00017; right angular gyrus: r = .554, p = 0.00018; left superior frontal gyrus (SFG): r = .564, p = 0.00014 and left medial frontal gyrus (MOG): r = .578, p = 0.00009. Peakvoxel, sub regions and cluster sizes are shown in table? 4.

**Figure 6:**
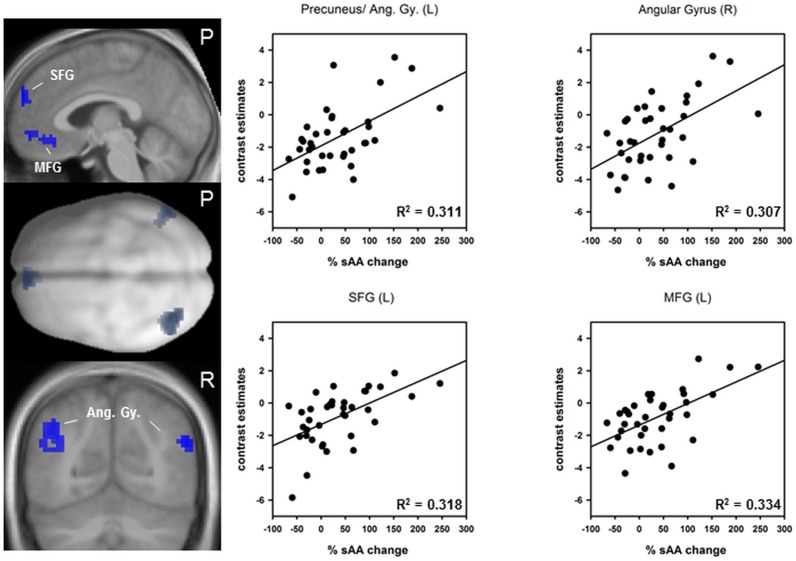
Conjunction of the task induced deactivation (TID) in run 1 and percentage sAA increase immediately prior to scanning. *Left:* upper and lower figures represent sagittal and coronal slices of an anatomical spatially normalized mean image of all subjects. The middle figure represents a dorsal view of a smoothed standard rendered brain. Colour blobs indicate regions that are deactivated during the task and influenced by relative salivary alpha-amylase (sAA) increase. *Right:* scatter plots and coefficient of determination R^2^. SFG: superior frontal gyrus; MFG: medial frontal gyrus, Ang. Gy: angular gyri.

**Table 4:** Peak voxel and sub regions from the conjunction analysis of the task induced deactivation (TID) during run 1 and relative sAA increase immediately prior to scanning.

L/R	Brain Region	T value	cluster size k	coordinates			r =	p =
				x	y	z		
L	Precuneus	4.38	132	–39	–70	34	.558	.00017
L	Angular Gy.	3.02		–54	–67	31		
L	MTG	3.05		–45	–61	22		
R	Angular Gy.	4.32	43	54	–67	28	.554	.00018
R	STG	3.27		48	–58	22		
L	SFG	4.10	51	–3	59	28	.564	.00014
L/R	SFG	2.97		0	50	19		
L	SFG	2.91		–9	53	40		
L	MFG	3.86	46	–6	35	–14	.578	.00009
L	MFG	3.75		–3	53	–8		

Peakvoxel from the conjunction analysis are based on a voxelwise p<0.005, uncorrected and a minimum cluster size of k = 30. MTG: middle temporal gyrus; STG: superior temporal gyrus; SFG: superior frontal gyrus; MFG: medial frontal gyrus.

As in the upper section, the conjunction analyses yielded no significant results during the second run.

#### Correlation between the extracted beta values and behavioural data

Reaction times (RT) negatively correlated with task induced activation in the left SMA: r = –.400, p<0.014, and with TID in the left angular gyrus r = –.410, p<0.012. In other words faster reaction times were associated with higher SMA activation and a lower suppression of left angular gyrus activity. Positive correlations could be observed between correct responses and task induced activation in the following structures: left SMA: r = .403, p<0.013, left lentiform nucleus: r = .374, p<0.022 and the right declive: r = .395, p<0.016. Scatter plots and coefficients of determination (R^2^) are presented in figure? 3b. No significant correlations with RTs or correct responses could be found for: the right angular gyrus, SFG, MFG, left precentral and left right lentiforn nucleus.

## Discussion

Using sAA as an indicator for sympathetic activation during an fMRI examination, the aim of the present study was to observe how sympathetic arousal is related to neural and behavioural data. Our fMRI protocol encompassed two experimental runs using the same paradigm in order to investigate the potential influence of scanner related sympathetic arousal on behavioural and neural responses at several time points of the scanning procedure. We were able to show that task induced activation in motor regions and the TID were affected by sympathetic arousal. Furthermore, sAA increases were associated with improved performance during scanning. These findings were however restricted to the first run.

### sAA changes

The sAA profile yielded the most pronounced increase immediately before subjects entered the scanner. After the peak sAA secretion remained at high levels during the first part of the experiment but decreased during the structural scan. A second rise of sAA secretion was observed at the end of the examination. Findings are in line with previous reports on heart rate changes during scanning [5]. It has been suggested that preparing the individual for the scan already bears a stressful component. Furthermore, it has been assumed that the initial observed increase of sympathetic markers prior to scanning can be attributed to the anticipation of the upcoming procedure [32]. This assumption was supported by changes in mood parameters [1] and the often reported pre-scan anxiety e.g. [4]. In spite of the strong sAA increase immediately before entering the scanner, only some (64.8%) but not all (35.1%) subjects react with an enhanced sAA secretion. This finding fits well with anxiety ratings in patients undergoing diagnostically MRI scanning. Results showed that 30–40% of the patients rated mild to severe anxiety whereas others rather seemed to be relaxed during the investigation [33]–[35].

The decrease of sAA secretion after the first part of the fMRI examination could be explained by habituation processes to the scanning environment [1], [21]. In another study conducted in our lab [2] we are able to show that subjective distress decreased continuously during the first 12 minutes of a fMRI investigation. Regarding the second increase at the end of the investigation, it might be possible that subjects may have aroused themselves after the structural scan. It has been also discussed by Chapman and colleagues [5] that the increase reflects the anticipation of the MRI session to end. Because MR staff informed the subjects that the fMRI session was finished before the last saliva sample was collected, it may be also possible that some movements after the scan but prior to saliva collections raised sAA levels.

### Conjunction of sympathetic arousal and task induced activation

The conjunction analysis yielded several regions that correlate with percentage increase of sympathetic activity. These effects could not be simply explained by sympathetic effects on vasoactivity, because these affect the whole brain vasculature but not particular structures. The analysis, however, has only yielded associations with specific parts of the entire network involved in motor preparation, performance and motor timing: [27], [36], [37] left SMA, left primary motor cortex left and right lentiform nuclei (which comprises the putamina and the globi pallidi) and the right cerebellar declive. Other regions like the middle occipital gyri or the superior parietal regions remained unaffected. These findings are in line with animal studies reporting higher responsiveness of motor neurons after immobilization-restraint [38] or stimulation of the locus coerulus-noradrenaline (LC-NA) system [39]. Furthermore, evidence from human fMRI studies revealed positive associations between motor regions and electrophysiological data (skin conductance, heart rate) during a stressful task [12], [13]. Due to the close interactions between NA and motor neurons [40] we conclude that the enhancing effects of NA on these motor areas account for the main behavioural differences during the first run. We therefore calculated the correlation between neural activation in motor areas and behavioural responses. The analyses clearly showed an association between the SMA and RTs as well as correlations between the SMA, left lentiform nucleus, the right declive and the accuracy data. However, it might be also possible that peripheral sympathetic reactions, for example an increased blood flow to musculature, additionally contributed to faster responses [10]. During the second run however, no associations between neural or behavioural data and percentage sAA changes could be observed. As outlined above, subjects habituate to the scanner environment over time. It is possible that the combination of attenuated sympathetic arousal and decreased subjective distress may explain the lack of results during run 2. Moreover, we discussed that the second increase at the end of the examination might be evoked by movements after the scan or by anticipating the end of the examination. Given this assumption, the second rise of sympathetic activity could not have an effect on task performance or neural correlates. Although our results clearly showed an influence of scanner related sympathetic arousal of neural and behavioural data during run 1, neither brain activity nor task performance differ significantly between run 1 and run 2. It is possible that the low mental effort acquired by the task may account for the lack of effects. Future studies are needed to clarify the effect of scanner related stress reactions on neural correlates of cognitive challenging tasks. However, an influence on behavioural and neural data during the first run was clearly shown. These results should be considered in further fMRI designs, in particular when motor tasks were used. It is also possible that pharmacological studies using adrenergic agents are affected by sympathetic effect especially during the beginning of the experiment.

### Conjunction of sympathetic arousal and TID

We observed regional conjunctions of the first sAA increase and several regions that were deactivated during task performance. Medial PFC and parietal regions (angular gyri and precunei) were known key nodes of the default mode network (DMN) that was supressed during active task performance [18], [41]. Our results show that, the higher the sympathetic reaction, the lower the deactivation in medial PFC and parietal brain areas. A study by Qin et al. [42] observed a decreased suppression after acute stress exposure in frontal brain regions similar to those in the present study (cp. Qin et al. [42]; supplementary material). It was discussed that a decreased suppression might reflect difficulties in inhibiting internal thoughts that are unrelated to the task but might be associated with experienced anxiety and distress. In the study by Qin and colleagues it was considered that those stress driven internal thoughts might impair goal directed behaviour. However, in the present study we observed an improvement of task performance probably due to the low mental effort of the task. Besides the mentioned involvement of the DMN functions on internal thoughts, there is the assumption of a sentinel function of the DMN. The sentinel function is to detect potentially bodily or environmental changes and was associated with a alertness and response readiness [43]. A reduced suppression in particular in parietal regions (angular gyri) during task performance could thus contribute to the improved behavioural reactions of aroused subjects. The observed negative correlations between RTs and left angular gyrus activity (comprising the left precuneus) clearly stressed this assumption. In other words, we here showed that a lower suppression of left angular gyrus activity correlates with faster responses. This result is in line with findings from animal studies showing that a high tonic NA release mode of the LC-NA system was associated with an improved detection of salient and unexpected stimuli. Moreover, the angular gyri have been shown to be anatomically connected to premotor regions [44]. The findings discussed in this section should be considered by studies investigating the TID during brain stimulation as well in investigations of resting state networks [45].

In addition, the positive association between the reported regions and sympathetic reactions corroborate findings from prior studies that showed associations between sympathetic arousal and task independent processes. After a median spilt procedure of high and low skin conductance responses (SCR) during a cognitive challenging task Zang and colleagues [11] observed task independent activations in the anterior cingulate and medial prefrontal regions in the high compared to the low SCR group. Associations between medial prefrontal activity and pupillary arousal responses were reported by Critchley et al. [10], [46]. It was discussed that these regions seem to be related to the regulation of stress and autonomic arousal. Interestingly the medial SFG has been shown to correlate negatively with the stress hormone cortisol 55 minutes after a laboratory stress exposure in a glucose PET study by Kern and colleagues [47]. It might be possible that the negative cortisol association with medial SFG regions reflects processes of stress termination long time after the stress protocol. This was quite different from the present study where the sympathetic arousal increased immediately prior to scanning.

### Limitations

Results, however, have to be interpreted in the methodological limits of the investigation. We cannot offer a direct control condition where subjects are subdivided either to a lab or a scanner session; we can nevertheless draw some within-subject conclusions based on the course of the entire experiment that included a 20 minute lab training prior to the scanner session. During this training no significant changes in sAA were found. SAA peaked for the first time immediately prior to scanning. This finding was supported by other studies that recorded sympathetic [5], [32] or subjective stress parameters [4], [6], [9], [33] Moreover, in another study we recently showed that subjective stress ratings were higher in a scanner compared to a laboratory setting [2].

Furthermore, only scanner naive subjects were included in the present study, it is possible that subjects habituate to the scanner environment after repeated participation in fMRI investigations which in turn prevented the observed behavioural and neural effects. However, in another study conducted in our lab, we were able to show that repeated fMRI scanning led to a habituation of subjective distress but increased the number of subjects showing a neuroendocrine response [2]. Thus repeated measurements can lead to either a habituation or sensitisation neuroendocrine reactions [2], [48].

Furthermore, we here used a very simple target detection task. Other functions like attentional inhibition [49] or cognitive flexibility [50] have been shown to be negatively influenced by elevated sympathetic arousal and stress. However, these investigations used study designs where first a stressor, then a cognitive task was presented. Stress reactions in these situations occur after stressor offset. These clearly differ from scanner related sympathetic reactions where the task was performed during the increase of the sympathetic arousal. As outlined by Elling et al., [51] the effect critically depends on the study design. For example, in the case of memory tasks, higher sympathetic arousal at the beginning of an fMRI investigation could positively influence encoding and consolidation of information followed by improved retrieval at later time points [52]–[54]. Contrary to the results of the present study, distress and arousal evoked by negative mood induction by highly aversive pictures or videos has also been shown to impair attentional processes [55] or working memory performance [42]. It might be possible that positive effects are specific for environmental treads like the confined space of the scanner bore or cognitive challenging tasks without negative emotional content. Future studies are needed to investigate effects of scanner related enhanced levels of arousal on other cognitive and affective functions.

Finally, it should be also taken into account that the observed effects may vary due to the population studied (e.g. age, psychiatric morbidity, familiarity with the scanner). Differences in sympathetic activity between groups could be misinterpreted as specific correlates of a disease or age related effects. Future studies are clearly needed to evaluate if patients and controls or different groups differ in sympathetic activation during fMRI examinations.

## Conclusions

We here demonstrated that sympathetic stress reactions correlate with behavioural data and neural activation patterns. These results are in line with prior findings but showed for the first time that sympathetic arousal elicited by the scanning session itself could account for such effects. It could be assumed that studies investigating adrenergic agents or patient groups are affected by this bias of scanner related arousal. As a consequence of high sympathetic arousal at the beginning of the scanning procedure, it might be advantageous to acclimatise the subjects to the scanner setting. Ten to 15 minutes last out in order to decrease sympathetic and subjective stress responses [1], [2]. Contrary to common practice to end with the structural scan, it is preferable to start the fMRI scanning sessions with the anatomical sequence. This can help to obtain more homogenous arousal levels prior to cognitive testing. Future studies are clearly needed to investigate how more homogenous arousal level of scanned subjects can be obtained. As outlined by Zandbelt, et al. [24] careful planning of the study and reduction of confounding factors enhances the statistical power of an experiment and could reduce sample size. Nevertheless, it is recommended to record physiological parameters and integrate them into the design model as covariates.
